# Phylogenomic insights into evolutionary trajectories of multidrug resistant *S. pneumoniae* CC271 over a period of 14 years in China

**DOI:** 10.1186/s13073-023-01200-8

**Published:** 2023-07-04

**Authors:** Yuan Zeng, Yuqin Song, Lanqing Cui, Qi Wu, Chao Wang, Adriano Cappellazzo Coelho, Gang Zhang, Dawei Wei, Chao Li, Jingren Zhang, Jacques Corbeil, Yun Li, Jie Feng

**Affiliations:** 1 State Key Laboratory of Microbial Resources, Institute of Microbiology, Chinese Academy of Sciences, Beijing, China; 2 College of Life Science, University of Chinese Academy of Sciences, Beijing, China; 3 Institute of Clinical Pharmacology, Peking University First Hospital, Beijing, China; 4 State Key Laboratory of Mycology, Institute of Microbiology, Chinese Academy of Sciences, Beijing, China; 5 Departamento de Biologia Animal, Instituto de Biologia, Universidade Estadual de Campinas (UNICAMP), Campinas, Brazil; 6 Center for Infectious Disease Research, Department of Basic Medical Science, School of Medicine, Tsinghua University, Beijing, China; 7 Department of Molecular Medicine, Big Data Research Centre, Nutrition Health and Society Centre (NUTRISS), INAF Institute Intelligence and Data, Laval University, Québec, Canada

**Keywords:** *Streptococcus pneumoniae*, Multidrug-resistant Clone, CC271, Vaccine escape, Whole-genome sequencing

## Abstract

**Background:**

*Streptococcus pneumoniae* is a gram-positive opportunistic pathogen, and infection risks of *S. pneumoniae* can be profoundly augmented by its acquired multidrug-resistance (MDR). The rapid development of MDR in *S. pneumoniae* was attributed to the international dissemination of a small number of multidrug-resistant “clones.” Clonal complex (CC) 271 is a prevalent MDR CC in the world and the most prevalent CC in China. However, the evolutionary trajectories of multidrug-resistant *S. pneumoniae* CC271 in China still are largely unknown.

**Methods:**

We investigated a collection of 1312 *S. pneumoniae* isolates collected from 28 tertiary hospitals in China from 2007 to 2020. Recombination prediction and recombination-masked phylogenetic analysis were combined to determine the population structure and mode of evolution of CC271. Data from the Global Pneumococcal Sequencing program (GPS) were combined to understand the global distribution of clones identified in this study. Bayesian analysis were recruited to analysis the evolutionary dynamics of dominant clones within CC271 in China.

**Results:**

The phylogenomic analysis resulted in the discovery of two globally distributed clones, ST271-A and ST271-B. ST271-A was a derivative of ST236 and an ancestor of ST271-B and ST320, refining the internal phylogenetic relationship of CC271. ST271-B was the most dominant clone in China, with higher β-lactam resistance especially for cephalosporins comparing to other MDR clones. Bayesian skyline plot showed a rapid expansion of 19F ST271-B from 1995 to 2000, which correlates with the widespread use of cephalosporins in the 1990s in China. 19A ST320, a vaccine-escape clone, is the second largest population in China. The Bayesian skyline plot showed that the 19A ST320 began to expand rapidly around 2001, which appeared to coincide with the prevalence of 19A after application of PCV7 in 2000 in the USA. We also observed frequent transmission of 19A ST320 between countries. It suggests that mass vaccination in some countries could affect the prevalence of clones in unvaccinated countries in the context of high-frequency international transmission.

**Conclusions:**

Our results refined the internal phylogenetic relationship of CC271, showing that the 19F ST271-B and 19A ST320 evolved independently from ST271-A, with different histories and driving forces for their evolution and dissemination in China.

**Supplementary Information:**

The online version contains supplementary material available at 10.1186/s13073-023-01200-8.

## Background

*Streptococcus pneumoniae* is a gram-positive opportunistic pathogen notorious for causing pneumonia, otitis media, meningitis, and bronchitis, resulting in significant morbidities and mortalities. *S. pneumoniae* remains the leading source of fatal infections in children aged < 5 years [1]. Disquietingly, infection risks of *S. pneumoniae* can be profoundly augmented by its acquired antimicrobial resistance (AMR), especially multidrug-resistance (MDR), defined as resistance to at least three classes of antibiotics [2]. Genotyping of multidrug-resistant pneumococci has suggested that the rapid development of MDR in *S. pneumoniae* could be attributed to the international dissemination of a small number of multidrug-resistant “clones” of closely related bacteria [3]. Therefore, introducing an evolutionary perspective to analyze the intraspecific evolutionary history of MDR in *S. pneumoniae* has become an essential and important approach.

Clonal complex (CC) 271 is one of the most widely distributed multidrug-resistant CC in the world, whose history has been investigated [4]. CC271 includes three well-known pandemic multidrug-resistant clones, sequence type (ST) 320, ST236, and ST271. Of these three, ST236 isolates were the original identification of CC271, which were first detected in Taiwanese hospitals in 1997 and named Taiwan19F-14 [5]. ST271 is a single locus variant (SLV) of ST236 and belongs to serotype 19F. ST236 and ST271 disseminated globally before the introduction of the 7-valent pneumococcal polysaccharide conjugate vaccine (PCV7) [6, 7]. Since the approval of PCV7 in the USA in 2000, the incidence of invasive pneumococcal disease (IPD) caused by vaccine serotypes, including 19F, was significantly reduced in countries with mass vaccination [8]. After that, ST320 with serotype 19A became a highly prevalent multidrug-resistant clone post-PCV7 in these countries, which was an SLV of ST271 and a double locus variant (DLV) of ST236 [9]. The 19A isolates were reported to be derived from the 19F isolates of ST320, most probably due to a recombination event resulting in a capsular switch [4, 10]. Therefore, the evolutionary relationship of these pandemic multidrug-resistant clones in CC271 could be due to locus variants, i.e., ST236 was more ancestral than ST271, and ST320 was derived from ST271 [11]. Nevertheless, such a scenario requires further verification by whole-genome-based phylogenetic analyses.

As mentioned earlier, in countries where PCV7 was extensively administered, the vaccine plays a central role in the dissemination and evolution of *S. pneumoniae*. Therefore, an intriguing question arises, i.e., what the evolutionary trajectories of *S. pneumoniae* will be in those countries where PCV7 was not widely used, and what will be the impact of vaccination on these countries? Considering the case in China as an example, PCV7 and PCV13 were licensed in China in 2008 and 2016, respectively, which was later than that in the USA (2000 and 2010, respectively). Since no pneumococcal vaccines were included in China’s National Immunization Program (NIP), PCV7 and its replacement have only been taken by a small fraction of the Chinese population [12]. Thus, vaccine serotypes such as 19F and 19A are still the most prevalent serotypes in China [13]. Therefore, it would also be of interest to study the evolutionary dynamics of prevalent vaccine serotypes in China. Genome sequencing is a cost-effective approach widely applied to decipher pathogens’ evolution and transmission routes [14, 15]. In this study, we conducted whole-genome sequencing on a set of 1,312 isolates of *S. pneumoniae* collected from 28 tertiary hospitals distributed in 18 provinces of China from 2007 to 2020 to gain insight into the pneumococcal population structure. By introducing genomic approaches combining phylogenetic and recombination analyses, we provide evidence on the comprehensive phylogenomic relationship of the clones of CC271 different from previous understanding. We found that the two most dominant clones in China, 19F ST271-B and 19A ST320, diverged separately from a recent common ancestor, 19F ST271-A. We further analyzed and speculated on their evolutionary dynamics and driving force of expansion. The high consumption of cephalosporins is the most likely reason for the prevalence of 19F ST271-B in China. In contrast, the prevalence of 19A ST320 appears to be impacted by not only its competitiveness such as antibiotic resistance but also its high intensity of global transmission. The results of our study provide insight into our understanding of the evolutionary trajectories of multidrug-resistant *S. pneumoniae* CC271 in China.

## Methods

### Genome sequencing and assembly

From 2007 to 2020, we obtained 1312 isolates of *S. pneumoniae* from 28 tertiary hospitals across 18 provinces in China as part of the China Antimicrobial Resistance Surveillance Trial (CARST) program [16]. The isolation information of these isolates was organized and summarized every 2 years. Genomic DNA was extracted using Wizard® Genomic DNA Purification Kit (Promega, Madison, USA). Samples were sequenced on an Illumina HiSeq 2000 platform with approximately 200 × coverage. Reads were assembled using SPAdes [17] (v3.13.1), and different lengths of k-mer (21,33,45,55,63,77) were used to obtain the optimal results with the fewest number of scaffolds.

### Antibiotic susceptibility test

The MICs of 13 antibiotics (penicillin, amoxicillin, cefuroxime, ceftriaxone, cefepime, imipenem, erythromycin, clindamycin, moxifloxacin, levofloxacin, vancomycin, chloramphenicol, and azithromycin) were determined using the agar dilution method [18]. The MICs were categorized into either susceptible, intermediate, or resistant according to CLSI (Clinical and Laboratory Standards Institute) 2021 guideline [19]. For β-lactam antibiotics, the breakpoint of parenteral (nonmeningitis) was used.

### Multilocus sequence typing and serotyping

Serotype was predicted in silico using SeroBA [20] (v1.0.0). The isolates were assigned STs by aligning seven housekeeping genes (*aroE*, *gdh*, *gki*, *recP*, *spi*, *xpt*, and *ddl*) to the MLST database [21] (https://pubmlst.org/organisms/streptococcus-pneumoniae) using BLASTn [22] (v2.11.0). The STs were clustered into CCs under single locus variant (SLV) criterion using goeburst [23] (v1.2.1). Any CC consisting of only two STs is called a doubleton, and any CC consisting of only one ST is called a singleton. For each antibiotic, a chi-square test or Fisher’s exact test of the scipy.stats package [24] in Python was selected according to the sample distribution to explore whether the proportion of nonsusceptible isolates of each CC was significantly higher than that of the 1312 isolates.

### Phylogenetic construction

The general feature format (gff) files of 1312 isolates produced by Prokka [25] (v1.14.6) were used as the input of Roary [26] (v3.13.0) to create a core gene alignment. A maximum-likelihood tree was constructed based on the alignment using RAxML [27] (v8.2.12) with the GTRGAMMA method.

The sequencing reads of 526 CC271 isolates in our samples were mapped to the complete genome sequence of Taiwan19F-14 (NC_012469.1) using Snippy [28] (v4.6.0). The prediction and removal of putative recombining regions were conducted using Gubbins [29] (v3.0.0) with the GTRGAMMA method. Then, a phylogenetic tree with ultrafast bootstrap values was reconstructed using IQtree [30–32] (v2.1.4-beta). Furthermore, 19F ST271 (*n* = 86) and 19A ST320 (*n* = 283) from the GPS database were included to reconstruct the global phylogeny of 19F ST271 (*n* = 387) and 19A ST320 (*n* = 458) using the same above-described method, respectively. Strain Taiwan19F-14 (19F ST236) was used to root the tree. For the phylogeny of 19A ST320, we changed the reference genome and outgroup into the isolate SP65 of 19F ST320 in our sample because 19F ST320 is the more recent ancestor of 19A ST320.

### Analysis of genotype and phenotype of β-lactam resistance

The alignments of PBP2b and PBP2x protein sequences of the 526 CC271 isolates in our sample were generated using MAFFT [33] (v7.475). Three isolates with incomplete PBP sequences were excluded. The phylogenetic trees of PBP2b and PBP2x sequences were constructed using IQTree.

For each of the six β-lactam antibiotics (penicillin, amoxicillin, cefuroxime, ceftriaxone, cefepime, and imipenem), the Wilcoxon test in the R package ggpubr [34] was performed to investigate whether the mean MIC among 19F ST271-A, 19F ST271-B, and 19A ST320 was significantly different. Due to the diversity of PBP2x and PBP2b sequences, the MICs for six 19F ST271-A and all 13 19F ST236 isolates in our data may not be representative and were not included in the comparison of MICs.

### Temporal phylogenetic reconstruction

A Bayesian coalescent analysis using Beast2 [35] (v.2.6.3) was conducted on the alignment of 301 19F ST271 isolates in our samples after the removal of recombining regions. The temporal signal was evaluated using TempEst [36] (v1.5.3) and examined using BETS [37]. A starting tree reconstructed using IQTree was used to fix the topology of the phylogeny. The optimal models and tree priror were determined using Path Sampling/Stepping-stone (PS/SS) [38, 39] analysis. The optimal substitution model was determined using bModelTest [40]. Parameters were estimated with an effective sample size (ESS) of > 200. We then reconstructed the evolutionary dynamics of 19F ST271 in China using the same method with the Coalescent Bayesian skyline as tree prior [41]. The same method was used for Bayesian skyline analysis of 175 isolates of 19A ST320.

### Trend test

The proportion of 19F ST271 or 19A ST320 isolates every 2 years was calculated. Significant upward or downward trends were examined using the Cochran–Armitage test in the DescTools package [42] in R.

## Results

### CC271 is the most prevalent clonal complex of multiple drug resistance in China

**Fig. 1:**
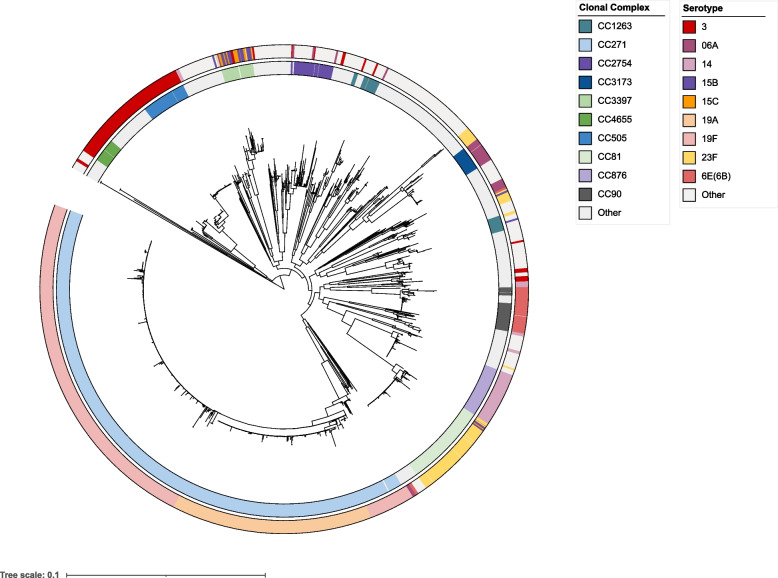
Molecular typing results and maximum likelihood tree of 1312 *S. pneumoniae* isolates. The tree was constructed based on 1100 core genes and midpoint-rooted. The color strips represent the clonal complex (CC) and serotype from inside out. The main CCs and serotypes are represented by distinct colors, while other CCs and serotypes are uniformly displayed in white

A total of 1,312 isolates of *S. pneumoniae* were collected from 2007 to 2020 in China (Fig. S1). The majority of the isolates were retrieved from sputum samples (929, 70.8%), and the remaining isolates were retrieved from blood samples, secretions, throat swabs, cerebrospinal fluid, drainage, and urine. The proportion of isolates from the elderly, adults, children, and unknown age group was 27.4%, 35.1%, 30.2%, and 7.3%, respectively. We sequenced the 1312 *S. pneumoniae* isolates on an Illumina HiSeq 2000 platform and constructed a phylogenetic tree based on the core genes of these 1312 isolates (Fig. 1). The largest cluster was represented by the isolates of CC271, accounting for 40.1%, indicating that CC271 was the most prevalent CC in China. The other clusters included 28 CCs, among which CC81, CC876, and CC505 also occupied significant proportions in the population, accounting for 5.9%, 3.6%, and 3.1% of isolates, respectively. A total of 1302 isolates were assigned to 64 serotypes, whereas the remaining 10 isolates were unknown serotypes or non-encapsulated. The prevalent serotypes were 19F (354, 27.0%), 19A (181, 13.8%), 03 (127, 9.7%), 23F (95, 7.2%), and 14 (59, 4.5%). In particular, 19F and 19A primarily belonged to CC271. The serotypes covered by the 13-valent pneumococcal conjugate vaccine (PCV13) comprised 77.7% of our collection. The overall rate of invasive pneumococcal disease (IPD) inicidence of the 1312 samples was 12.8%. Notably, serotypes 14 and 09 V exhibit high rates of IPD incidence at 36% and 33%, respectively. CC876 exhibit a high rate of IPD incidence at 33%. We determined the minimum inhibitory concentrations (MICs) of 13 antibiotics for the 1312 isolates. Nonsusceptible rates for macrolides (erythromycin and azithromycin) and lincosamide (clindamycin) antibiotics were very high, more than 90% (Fig. S2). However, most isolates were susceptible to fluoroquinolones, and all isolates remained susceptible to vancomycin. The nonsusceptibility rates to different β-lactam antibiotics in the pneumococcal isolates ranged from 11% for penicillin to 65% for cefuroxime. We also examined the contribution of diverse CCs to the resistance of the entire population. We identified CCs with significantly higher non-susceptible rates to at least one of the 13 antibiotics than the average rates of all isolates, as shown in Fig. S3. CC271, the most successful pneumococcal CC in China, exhibited higher non-susceptible rates to multiple antibiotics than other CCs, especially to six β-lactam antibiotics, indicating that CC271 is a multidrug-resistant clonal complex and its prevalence would account for the high prevalence of MDR in China. Furthermore, CC81, CC3173, CC90, CC3397, CC876, CC2758, CC902, and a doubleton demonstrated significantly higher levels of resistance to one to three antibiotics than the average nonsusceptible rates of all isolates, separately.

### Phylogenomic analysis refines intra-clonal links of *S. pneumoniae* CC271

**Fig. 2:**
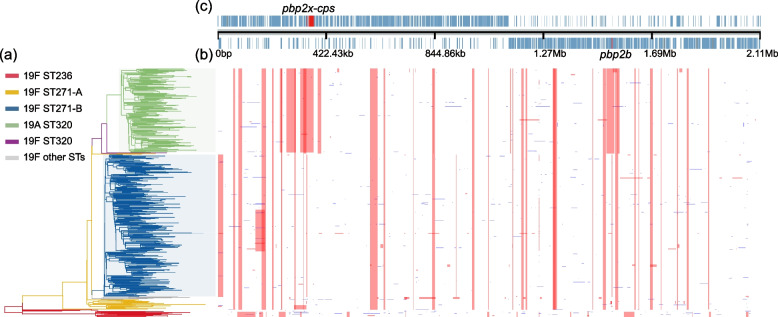
Phylogenetic tree and predicted recombination events of 526 CC271 isolates in China. **a** Recombination-masked WGS phylogeny; colors of the branches correspond to groups, which consist primarily of isolates with the corresponding ST and serotype. Red represents 19F ST236 (including Taiwan19F-14), orange represents 19F ST271-A, blue represents 19F ST271-B, purple represents 2 19F ST320 isolates, green represents 19A ST320, and gray represents a group consisting of other rare STs that are not focused. Blue and green shaded box represents the monophyletic group of 19F ST271-B and 19A ST320. **b** The predicted recombinations in CC271 isolates. Column corresponds to location in the reference genome, and row corresponds to taxon in the phylogeny. Red blocks represent putative recombination events shared by multiple isolates through common descent, and blue blocks represent putative recombination events occurred in a single isolate. **c** Annotation of the *S. pneumoniae* Taiwan19F-14 reference genome. Each block represents a coding sequence. Coding sequences of *pbp2x*, *cps* locus, and *pbp2b* are colored in red. *pbp2x* is located upstream of *cps* locus

To understand the overall population structure and mode of evolution of CC271 *S. pneumoniae*, we first aligned the read pairs of 526 CC271 isolates in our collection against the complete reference genome of *S. pneumoniae* Taiwan19F-14, which is considered as the original identification of CC271. Recombinations were predicted using Gubbins [29]. A maximum-likelihood phylogenetic tree was reconstructed after the removal of recombination regions (Fig. 2a). The tree was midpoint-rooted. According to tree topology and predicted recombination events of isolates, we proposed that the CC271 could be divided into five clones: 19F ST236, 19F ST271-A, 19F ST271-B, 19F ST320, and 19A ST320 (Fig. 2a). 19F ST271-B and 19A ST320 were predominant in CC271 of our collection, representing 289 and 175 isolates, respectively. The clone of 19F ST236 including Taiwan19F-14 was located at the root position, which was speculated to be the ancestor of the CC271 in previous study [11]. According to the MLST results, previous studies have favored the a priori assumption that ST320 is a derivative of ST271. Nonetheless, the presence of 19F ST271-A and 19F ST271-B provided new insights into the evolutionary trajectories among clones in CC271. Multiple 19F ST271 clades named as 19F ST271-A and located near a sister clone of 19F ST236. The branches corresponding to the emergence of 19F ST271-A preceded 19F ST271-B and ST320, which form two separate monophyletic groups, suggesting that 19F ST271-B and ST320 have independently emerged from 19F ST271-A. Accordingly, we refined the evolutionary relationships among clones in CC271.

The recombination analysis also supported the evolutionary trajectories among clones of CC271 (Fig. 2b, c). A total of 28 recombination events were shared by 19F ST271-A, 19F ST271-B, 19F ST320, and 19A ST320, but not by 19F ST236. In addition, four recombination events were exclusively detected in 19F ST271-B and 19A ST320 isolates, respectively. Among the four recombination events unique to 19F ST271-B, two were detected in penicillin-binding protein genes (*pbps*), *pbp2x* and *pbp2b*, and one was a large fragment deletion (15.7 kbp, corresponding to 3157–22,610 bp of the genomes of Taiwan19F-14) (Fig. 2b, c). Regarding the ST320, 19F ST320 underwent six additional recombination events relative to the 19F ST271-A. The recombination events in *pbp2x* and *pbp2b* were also detected, introducing different alleles. The 19A ST320 contained these six recombination fragments, but further underwent four unique recombination events. Among four recombinations, a recombination in 19A ST320 resulted in the replacements of *pbp2x* gene and the adjacent *cps* locus, which was reported to be associated with serotype switches from 19F to 19A, and the new *pbp2x* was reported to confer a similar β-lactam resistance level to that of 19F ST320 [4, 43].

### Characterization of penicillin-binding protein (PBP) and resistance phenotypes of clones in CC271

**Fig. 3:**
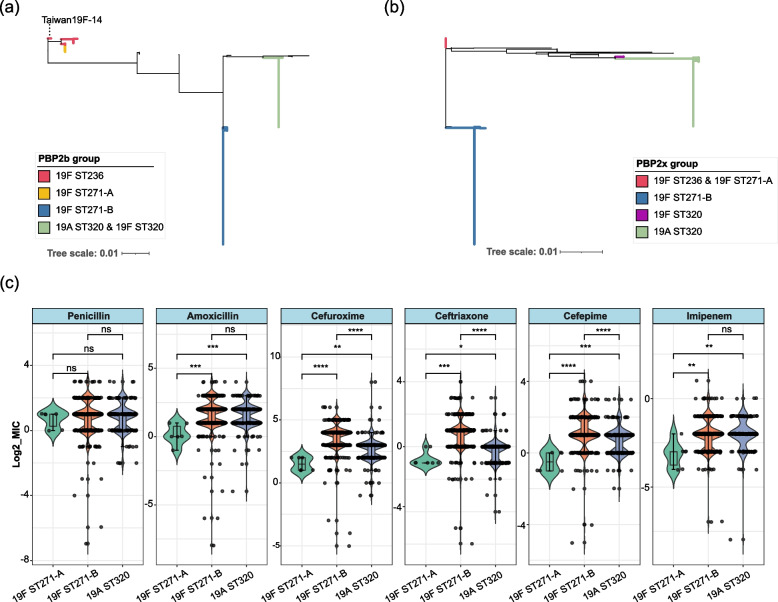
Phylogenetic tree of PBP amino acid sequences and β-lactam resistance phenotype comparison of groups in CC271. **a**, **b** Phylogeny of PBP2b (**a**) and PBP2x (**b**) amino acid sequences of 526 CC271 isolates. Bolded branches with colors are the manually selected representative sequences that correspond to each group and are identical or less different, and a few black branches that are not bolded represent PBP sequences with distinct variation that may be due to additional recombination and are not focused here. PBP2b sequence of Taiwan19F-14 (marked in figure) reference genome is similar but different from those of 19F ST236 isolates in our sample. All 19F ST271-A isolates had 100% identical PBP2b sequences with two 19F ST236 isolates in our sample. PBP2b sequences of 19F ST320 and most 19A ST320 were 100% identical. PBP2x sequences of most 19F ST236 and 19F ST271-A were 100% identical. **c** MICs of each β-lactam antibiotic in each group. The *X*-axis corresponds to each group, and the *Y*-axis corresponds to the log2(MIC) value. The presence of a significant difference between the two groups is marked in the figure, respectively. *p < 0.05. **p < 0.01. ***p < 0.001. ****p < 0.0001, ns, not significant

We conducted detailed analyses to detect the recombination of the three *pbp* genes primarily associated with β-lactam resistance in five clones [44]. The amino acid sequences of PBP1a were found to be 100% identical in all the CC271 isolates. Therefore, we used the amino acid sequences of PBP2x and PBP2b of CC271 isolates to construct the maximum-likelihood tree (Fig. 3a, b). The PBP2b amino acid sequence was clustered into three groups. The sequences of 19F ST236 and 19F ST271-A was clustered, and there were two 19F ST236 isolates possess identical PBP2b sequences to 19F ST271-A. All isolates of 19F ST271-B contain identical PBP2b, and 19F ST320 and 19A ST320 clustered and contain identical PBP2b. The sequences of PBP2X were clustered into four groups: 19F ST236 shared identical PBP2x with 19F ST271-A, and the sequences within each of the 19F ST320, 19A ST320, and 19F ST271-B were almost identical and distinguished from each other. This result is consistent with above mentioned description of the recombination of *pbp2x* and *pbp2b*.

To elucidate the resistance level of *S. pneumoniae* isolates belonging to different clones, we analyzed the determined MICs of six β-lactam antibiotics for 19F ST271-A, 19F ST271-B, and 19A ST320. Remarkably, we detected significant elevations in the MICs of amoxicillin, cefuroxime, ceftriaxone, cefepime, and imipenem for 19F ST271-B and 19A ST320 isolates compared with the MIC for 19F ST271-A, indicating that 19F ST271-A as an ancestral clone had less advantage in β-lactam resistance compared with its subsequent clones. Moreover, the MICs of three cephalosporins for 19F ST271-B were significantly higher than for 19A ST320. This may be partially due to the PBP2x amino acid sequence of 19F ST271-B containing three unique substitutions, M339F, M400T, and Y595F, compared with other clones in CC271 [45].

### Spatial and temporal analysis of population diffusion of 19F ST271

To understand the global distribution of 19F ST271-A and 19F ST271-B, we combined 19F ST271 in our collection with 75 19F ST271 isolates from the Global pneumococcal sequencing project (GPS) [46]. The isolates from GPS were from Africa (*n* = 50), Asia (*n* = 19, 16 from China), Americas (*n* = 4), and Europe (*n* = 2). The 387 genomes of 19F ST271 isolates formed two distinct clades (Fig. S4). One clade includes 19F ST271-A and isolates mainly from Africa which formed a relatively independent monophyletic clade adjacent to 19F ST271-A. Five of the six isolates from Europe and the Americas and four isolates from Asia (not including China) clustered with 19F ST271-A. Furthermore, the three PBPs (1a, 2b, 2x) of 11 isolates from Africa and three isolates from the Americas share 100% identical sequences with 19F ST271-A. The observation strongly suggests that 19F ST271-A-related isolates circulated broadly worldwide, while only one isolate from Europe and one isolates from Asia (not including China) clustered with 19F ST271-B.

**Fig. 4:**
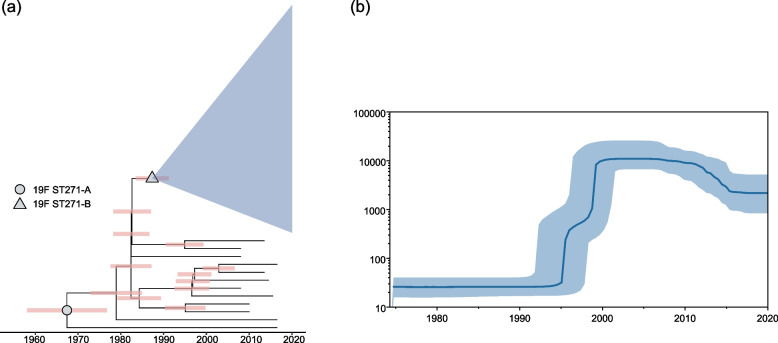
Bayesian phylogenetic analysis of 19F ST271 in China. To reduce the error from various sources, we used only the genomes of 19F ST271 (*n* = 301) from our collection. **a** Time-scaled phylogeny of 301 19F ST271 genomes. Nodes of the respective tMRCA (the most recent common ancestor) of 19F ST271-A and 19F ST271-B are marked with circle and triangle, respectively. The pink strip on each node represents a 95% confidence interval derived from highest posterior density (HPD) analysis for its differentiation time, corresponding to the timeline at the bottom of the figure. Clade of 19F ST271-B (*n* = 289) is collapsed and represented by blue-shaded triangle. **b** Bayesian skyline plot of genetic diversity shows sharp expansion of 19F ST271 during the 1990s. The vertical axis shows the estimated effective population size at the corresponding time. The shaded blue areas on either side of the line represent 95% HPD confidence intervals

To infer the temporal patterns within 19F ST271, we performed Bayesian phylogenetic reconstruction using BEAST2 under a Strict Clock model with a Constant population distribution using isolates from our collection. The TempEst root-to-tip plot shows a positive correlation between genetic distance and sampling year (Fig. S5), and the Bayesian evaluation of temporal signal (BETS) provided significant evidence of a molecular clock. Results showed that 19F ST271-B originated around 1988 (95% highest posterior density [HPD] interval 1983–1991), and 19F ST271-A appeared around 1967 (95% HPD interval 1958–1976) (Fig. 4a), with a median molecular clock rate of 6.55 × 10^−7^ substitutions per site per year (95% HPD interval of 5.71 × 10^−7^ to 7.37 × 10^−7^), lower than previously calculated values^4^. Using the Coalescent Bayesian Skyline population distribution, an almost same result is obtained that 19F ST271-B was estimated to originated around 1988 (95% HPD interval 1983–1991) and 19F ST271-A appeared around 1963 (95% HPD interval 1953–1975) (Fig. S6). We next conducted a Bayesian skyline analysis to determine the evolutionary dynamics of 19F ST271 and observed a very sharp rise during the period from 1995 to 2000 in China (Fig. 4b). We hypothesized that the rapid expansion of 19F ST271 correlates with the widespread use of cephalosporins in the 1990s in China and the advantage of 19F ST271-B in resistance for cephalosporins [47].

### Spatial and temporal analysis of population diffusion of 19A ST320

**Fig. 5:**
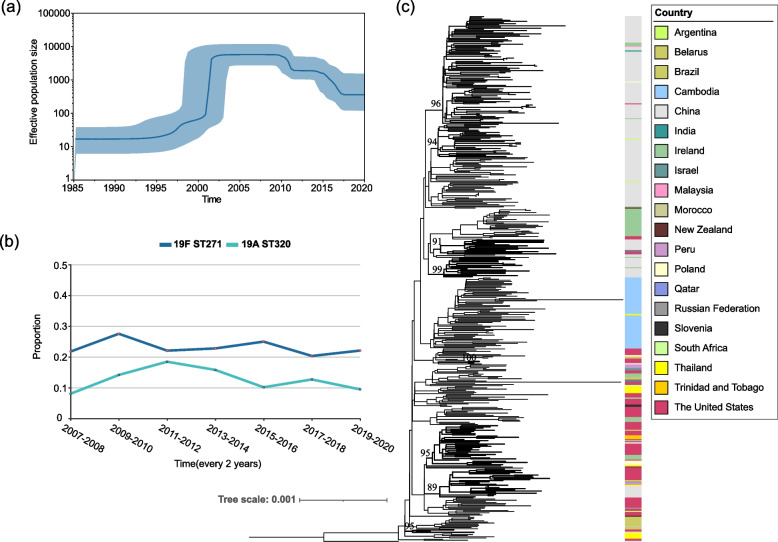
Phylogeny of 458 19A ST320 genomes from different countries, and temporal trends of 19F ST271 and 19A ST320 in China. **a** Bayesian skyline plot of genetic diversity shows sharp expansion of 19A ST320 around 2001. The vertical axis shows the estimated effective population size at the corresponding time. The shaded blue areas on either side of the line represent 95% HPD confidence intervals. **b** The proportion of 19F ST271 and 19A ST320 in our collection every 2 years. **c** Reconstructed recombination-masked phylogeny 19A ST320 genome, consists of samples from this study (*n* = 175) and GPS (*n* = 283). One of the 19F ST320 isolates in this study SP65 was used as outgroup. The color strip represents the country from which the sample was isolated. Bold clades include isolates from both China and the other countries, supported by ultrafast bootstrap values of ≥ 89, with the values marked on the nodes

In order to understand the evolution and dissemination of 19A ST320 in China, we performed Bayesian skyline analysis based on 175 isolates of 19A ST320 in our dataset. The results showed that 19A ST320 increased slightly around 1997 and sharply rose around 2001 in China (Fig. 5a), then decreased after 2010. We also calculated the proportions of 19A ST320 and 19F ST271 based on all *S. pneumoniae* isolates isolated in the corresponding years in our collection (Fig. 5b). The proportion of 19A ST320 showed a statistically significant increase from 2007–2008 to 2011–2012 (Cochran–Armitage test, *Z* = 2.62, *p* = 0.0087) and decreased after 2011–2012 (*Z* =  − 2.51, *p* = 0.012), while the proportion of 19F ST271 did not show statistically significant variation.

In order to understand the relationship between the 19A ST320 distributed in China and the world, we performed phylogeny reconstruction that included 458 genomes of 19A ST320 isolates from our collection and GPS. The dataset primarily consisted of isolates from China (184, 56.4%) and the USA (93, 28.5%). One isolate, SP65 from 19F ST320 in our collection, was used to root the phylogeny (Fig. 5c). The rooted phylogeny showed that most isolates of 19A ST320 in the clades close to the root were isolated from the USA, while most of the isolates from China clustered near the terminal branches of the tree. The phylogeny pattern demonstrated that some isolates of 19A ST320 from the USA diverged earlier than those prevalent in China, suggesting their ancestral position. In addition, eight subclades supported by high ultrafast bootstrap values (ranging from 89 to 100) consisted of isolates from both China and other countries. Among these subclades, six contained isolates from both China and the USA, implying frequent and widespread global transmission of 19A ST320.

## Discussion

Genome sequencing is a powerful approach to answering key issues, such as population dynamics, evolutionary trajectory, transmission route, and geographical origin. As an early example, the GPS project has demonstrated its strengths for pathogen genomic surveillance. It provided extensive information to understand evolution and spread of MDR clones, track vaccine-evading isolates, and advance genome-based characterization [48]. The immunization rates for the *S. pneumoniae* vaccine are low in China [12, 49], and there exists a heavy burden of pneumonia and meningitis caused by *S. pneumoniae* in China [50]. Unfortunately, the isolates from China represented only 3.74% of the GPS database. Furthermore, two recent reports on large-scale genomic analysis of *S. pneumoniae* were collected from China. One study included 128 *S. pneumoniae* isolates isolated from children aged < 5 years in Zhejiang, China, from 2009 to 2019, and the other study included 124 isolates isolated from children living in southwest China during 2017–2019 [51, 52]. Our study included 1312 *S. pneumoniae* isolates collected from 28 tertiary hospitals in 18 provinces of China over 14 years and provided a comprehensive insight into population genomics in pneumococcal epidemiology. We found that CC271 is the most dominant MDR clone complex in China, accounting for the β-lactam antibiotic resistance of pneumococci in China.

**Fig. 6:**
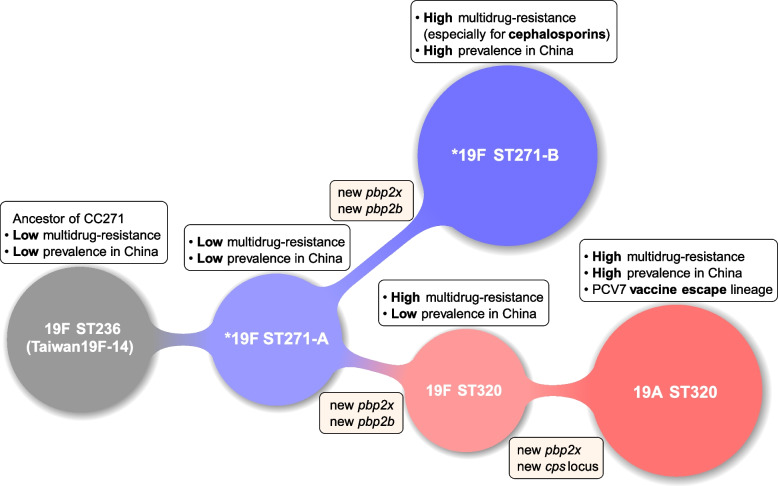
Schematic diagram of the evolutionary history of main members of CC271. Clones were represented by circles. Each junction represents a step of differentiation. The annotations above each circle show the characteristics of each clone. Annotations at each junction show changes in *pbp2x* and *pbp2b* genes or *cps* locus that occur during the process of differentiation. *Two global distributed clones of 19F ST271 distinguished in this study

With additional representative data, genomic analyses have the potential to offer a clearer picture of the evolution and global spread of pathogens [48]. Previous studies based on genotyping have speculated on the evolutionary trajectory of clones in CC271, from ST236 to ST271, followed by ST320 [11]. The evidence obtained in our study through phylogenetic reconstruction and recombination analysis of a temporally and geographically broad collection of genomes not only supports this hypothesis on the evolutionary relationship of clones in CC271 but also provides a more detailed evolutionary trajectories of clones in CC271 (Fig. 6). According to serotype, tree topology and predicted recombination events of isolates, we proposed that the CC271 should be divided into five clones. We identified two distinct clones of ST271 with serotype 19F, 19F ST271-A and 19F ST271-B. Multiple early differentiated 19F ST271-A clades reflecting an ancestral genotype from which two dominant descendants, 19F ST271-B and ST320, were derived independently. We proposed several characteristics based on our data that could help to recognize 19F ST271-A: it belongs to serotypes 19F and ST271, inherited identical PBP2b, PBP2x, and PBP1a sequences from 19F ST236 and further underwent 28 recombination events, did not obtain the dominant genotype (e.g., 4 recombination events) of 19F ST271-B, and had a significantly lower level of β-lactam resistance. Samples from the GPS database revealed that 19F ST271-A had been distributed in multiple continents; a significant number of isolates from multiple continents were clustered with 19F ST271-A and shared the same PBP2b, PBP2x, and PBP1a sequences, indicating that it had been widely spread worldwide, although it is not currently prevalent in China. Therefore, our results suggest that the 19F ST271-A genotype reported in this study is a starting point for the recent global epidemic of CC271, from which a variety of dominant or unprevalent genotypes have been derived.

19F ST271-B contained two recombination events, imported *pbp2x* and *pbp2b* alleles, compared with 19F ST271-A. The data collected by the US Active Bacterial Core Surveillance Program (ABC) during 1998–2013 demonstrated that the predominant resistance-associated PBP transpeptidase profile of 19F ST271 in the USA was consistent with that of 19F ST271-B [6]. Penicillin-nonsensitive isolates of 19F ST271 previously reported in the Czech Republic and Hong Kong, China, also had identical PBP1a, PBP2x, and PBP2b sequences with 19F ST271-B [53, 54]. These data suggest that 19F ST271-A and 19F ST271-B could be widely distributed worldwide. 19F ST271-B is currently the most dominant multidrug-resistant population of *S. pneumoniae* in China and has the highest resistance to multiple β-lactam antibiotics among CC271 clones. The Bayesian analysis speculated that 19F ST271-B originated around 1988, earlier than 1997 when the early ancestor of CC271, Taiwan19F-14, isolated, suggesting that the time of clonal differentiation could be earlier than the discovery of Taiwan19F-14. The Bayesian skyline analysis demonstrated a rapid expansion of 19F ST271-B during 1995–2000 in China, which correlates with the widespread use of cephalosporins in the 1990s in China, and consistent with the advantage of 19F ST271-B in resistance against cephalosporins [47].

The serotype 19A ST320 genotype has been highly successful in the US post-PCV7 [55], whereas 19A ST320 is the second largest population in our dataset, which shared the identical recombination sequence causing capsule switch to 19A ST320 in the USA [4]. The result of Bayesian skyline analysis speculated that 19A ST320 began to expand rapidly in China around 2001, shortly after PCV7 was licensed in the USA in 2000. This is consistent with previous reports that serotype 19A was rarely detected in China before 2000 [56]. By 2005 and 2006, the vaccine escape serotype 19A emerged as a major serotype in China and the USA [57, 58]. However, PCV7 was not licensed in China until 2008. Meanwhile, 19F ST320 has very similar resistance level to 19A ST320 in previous report and our data [4], but it has never been prevalent in China, suggesting that resistance phenotype could be the necessary but not sufficient condition to their prevalence. Phylogenetic analysis revealed the existence of multiple subclades shared between China and other countries. We proposed that the rapid expansion of 19A ST320 in China could associate with global epidemic and transmission, which may have increased the probability of initiation of expansion, while the main driving force during expansion could still be due to competitiveness, including antibiotic resistance. In addition, both Bayesian skyline analysis and the proportion of 19A ST320 in our sample demonstrated a significant decrease after 2011. However, PCV13 was not approved in China until 2016. PCV13 was licensed in the USA in 2010, and the incidence of IPDs caused by PCV13/non-PCV7 serotypes (primarily 19A and 7F) decreased significantly in children and adults in 2010 and 2011, respectively [59]. The application of vaccination affects the global prevalence of clones and may also indirectly affect countries without appropriate vaccination.

## Conclusions

Our study provides a large-scale and time-continuous *S. pneumoniae* genomic surveillance in China, cataloging the distribution of serotypes and occurrence of antibiotic resistance of *S. pneumoniae*. This type of study offers an evidence-based tool to inform future vaccine strategies. More representative data fill the gap in evolution and the global spread of different multidrug-resistant clones in CC271. 19F ST271-A was identified as an ancestral clone of 19F ST271-B and ST320. 19F ST271-B exhibited higher resistance to multiple β-lactam antibiotics, and its widespread dissemination is responsible for the multiple resistance of *S. pneumoniae* in China. The widespread of 19F ST271-B may be related to the high consumption of cephalosporins in China in 1990s. The dynamics of 19A ST320 in China correlated with the shift of 19A caused by mass vaccination in other countries, providing strong evidence that mass vaccination can indirectly affect countries where immunization was not concomitantly implemented. This is an argument in favor of global vaccination programs.

## Supplementary Information

**Additional file 1:** **Figure S1.** Sampling distribution of 1312 *S. pneumoniae* isolates in China. **Figure S2.** Proportion of each resistance category of 1312 *S. pneumoniae* isolates to 13 antibiotics. **Figure S3.** CCs with significantly higher nonsensitive rates to at least one of the 12 antibiotics than the average of all samples. **Figure S4.** Reconstructed Recombination-masked phylogeny of 387 19F ST271 genomes. **Figure S5.** Screenshot of TempEst root-to-tip plot of 301 19F ST271 isolates in China. **Figure S6.** Time-scaled phylogeny of 301 19F ST271 genomes with Bayesian skyline plot as tree prior.

**Additional file 2:** **Table S1.** The accessions of 1312 *S. pneumoniae* genomes deposited in the National Microbiology Data Center.

## Data Availability

Genomic sequence data used in this study have been deposited into the National Microbiology Data Center (NMDC; https://nmdc.cn/en); the accessions of each genome could be found in Table S1. Genomic sequence data were also deposited in GenBank of The National Center for Biotechnology Information (NCBI) under Bioproject PRJNA860820. The GPS dataset analyzed during the current study are available at: https://data.monocle.sanger.ac.uk/ [46].

## Abbreviations

CC: Clonal complex
ST: Sequence type
GPS: Global Pneumococcal Sequencing program
PCV: Pneumococcal polysaccharide conjugate vaccine
AMR: Antimicrobial resistance
MDR: Multidrug-resistance
SLV: Single locus variant
DLV: Double locus variant
IPD: Invasive pneumococcal disease
NIP: National Immunization Program
MIC: The minimum inhibitory concentrations
PBP: Penicillin-binding protein
BETS: The Bayesian evaluation of temporal signal
ABC: The US Active Bacterial Core Surveillance Program
CLSI: The Clinical and Laboratory Standards Institute
