# Supplementary material for: Phylogenomic insights into evolutionary trajectories of multidrug resistant S. pneumoniae CC271 over a period of 14 years in China
Source: Genome Med. 2023 Jul 4;15:46. doi: 10.1186/s13073-023-01200-8 (PMC10318735; doi:10.1186/s13073-023-01200-8)
Supplement: Supplementary file 1 — Additional file 1: Figure S1. Sampling distribution of 1312 S. pneumoniae isolates in China. Figure S2. Proportion of each resistance category of 1312 S. pneumoniae isolates to 13 antibiotics. Figure S3. CCs with significantly higher nonsensitive rates to at least one of the 12 antibiotics than the average of all samples. Figure S4. Reconstructed Recombination-masked phylogeny of 387 19F ST271 genomes. Figure S5. Screenshot of TempEst root-to-tip plot of 301 19F ST271 isolates in China. Figure S6. Time-scaled phylogeny of 301 19F ST271 genomes with Bayesian skyline plot as tree prior. [file 13073_2023_1200_MOESM1_ESM.pdf]

Phylogenomic analysis refines *Streptococcus pneumoniae* intra-clonal lineage links and reveals the impact of vaccination on international transmission

Yuan Zeng[1,2,*], Yuqin Song[1,*], Lanqing Cui[3,*], Qi Wu[4], Chao Wang[1], Adriano Cappellazzo Coelho[5], Gang Zhang[1], Dawei Wei[1], Chao Li[1], Jingren Zhang[6], Jacques Corbeil[7], Yun Li[3,#], Jie Feng[#]

[1] State Key Laboratory of Microbial Resources, Institute of Microbiology, Chinese Academy of Sciences, Beijing, China

[2] College of Life Science, University of Chinese Academy of Sciences, Beijing, China

[3] Institute of Clinical Pharmacology, Peking University First Hospital, Beijing, China

[4] State Key Laboratory of Mycology, Institute of Microbiology, Chinese Academy of Sciences, Beijing, China

[5] Departamento de Biologia Animal, Instituto de Biologia, Universidade Estadual de Campinas (UNICAMP), Campinas, Brazil

[6] Center for Infectious Disease Research, Department of Basic Medical Science, School of Medicine, Tsinghua University, Beijing, China

[7] Department of Molecular Medicine, Big Data Research Centre Nutrition Health and Society centre (NUTRISS), INAF Institute Intelligence and Data, Laval University

[*] These authors contributed equally to this work.

[#] Corresponding authors:

Jie Feng (Email: fengj@im.ac.cn; ORCID: 0000-0001-5172-6643)

Yun Li (Email: liyun03602@pkufh.com; ORCID:0000-0003-1173-1282)

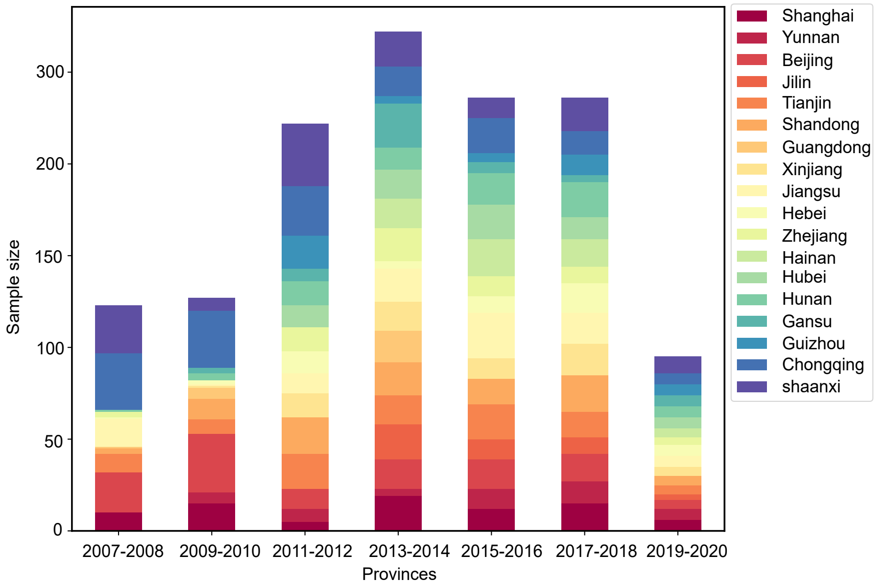

Figure S1. Sampling distribution of 1312 *S. pneumoniae* isolates in China. The number of samples isolated every 2 years, and the color corresponds to each province.

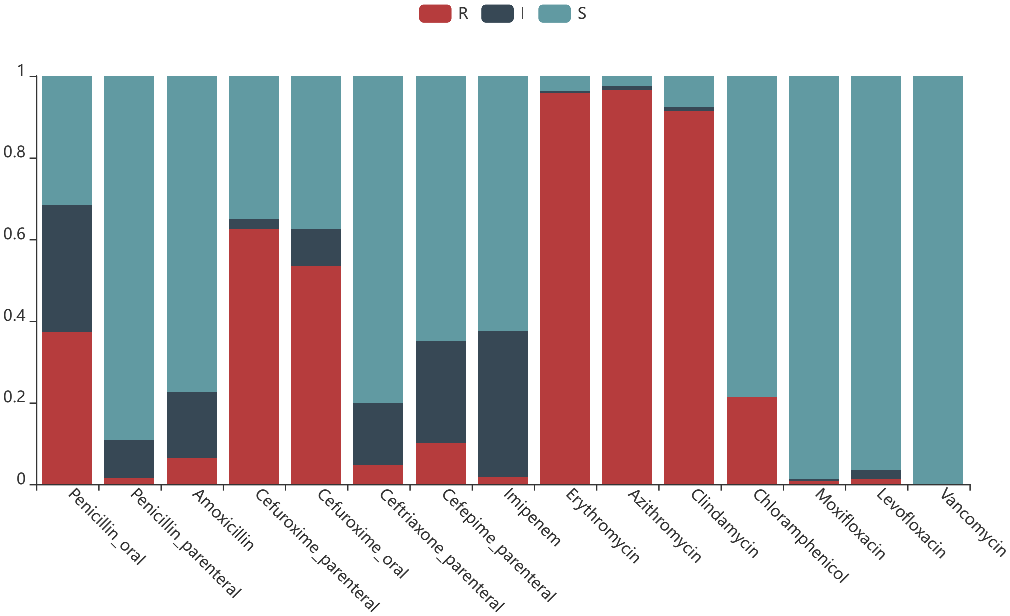

Figure S2. Proportion of each resistance category of 1312 *S. pneumoniae* isolates to 13 antibiotics. Y-axis shows the proportion of three resistance category: resistance (R), intermidate (I) and susceptible (S). The X-axis labels show the antibiotic names and the CLSI non-meningitis breakpoint used (oral or parenteral).

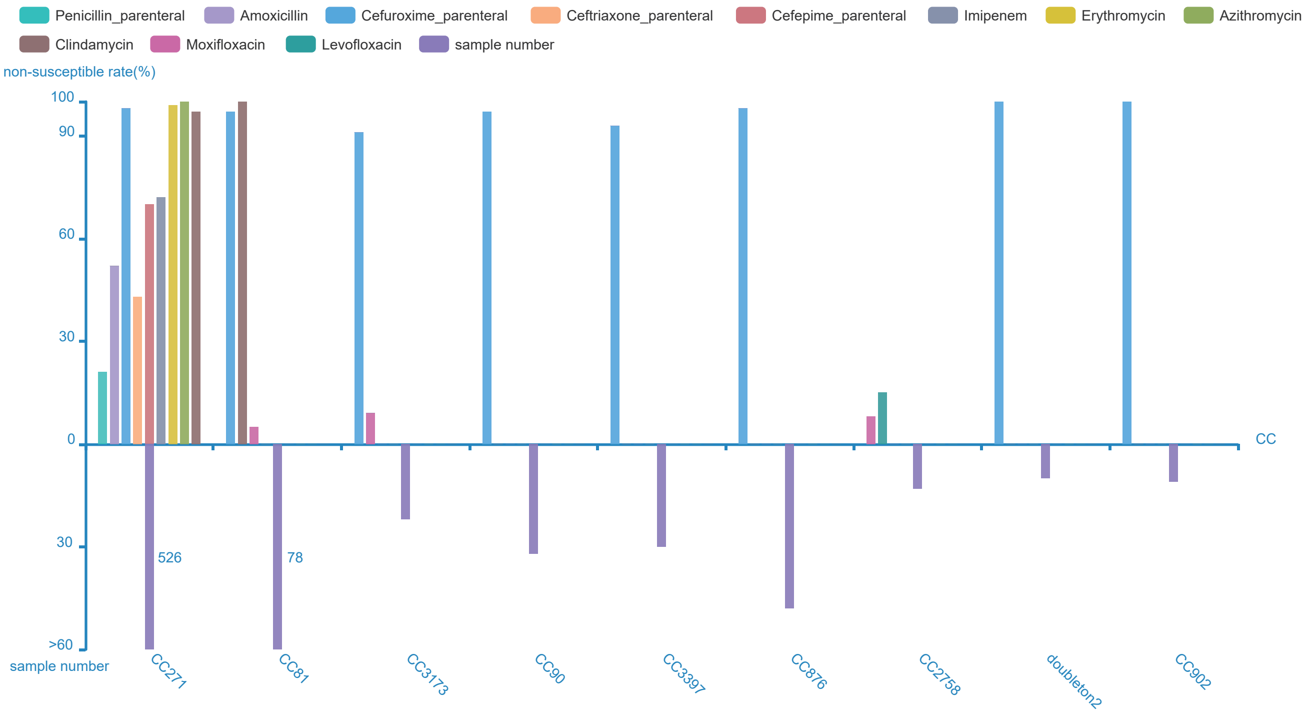

Figure S3. CCs with significantly higher nonsensitive rates to at least one of the 12 antibiotics than the average of all samples. The X-axis corresponds to each CC. The positive axis of the Y-axis represents the proportion of samples that were nonsensitive to each antibiotic, and each color represents an antibiotic. Each column indicates that the corresponding nonsensitive rate of CC to the corresponding antibiotic is significantly higher than the average nonsensitive rate to that antibiotic for all 1312 *S. pneumonia* isolates. In short, the number of columns reflects the overall level of multidrug resistance of a CC. All samples are sensitive to vancomycin and are therefore not shown. The negative axis of the Y-axis represents the sample size of the CC; if the sample size was >60, the specific number of sample size is marked in the figure.

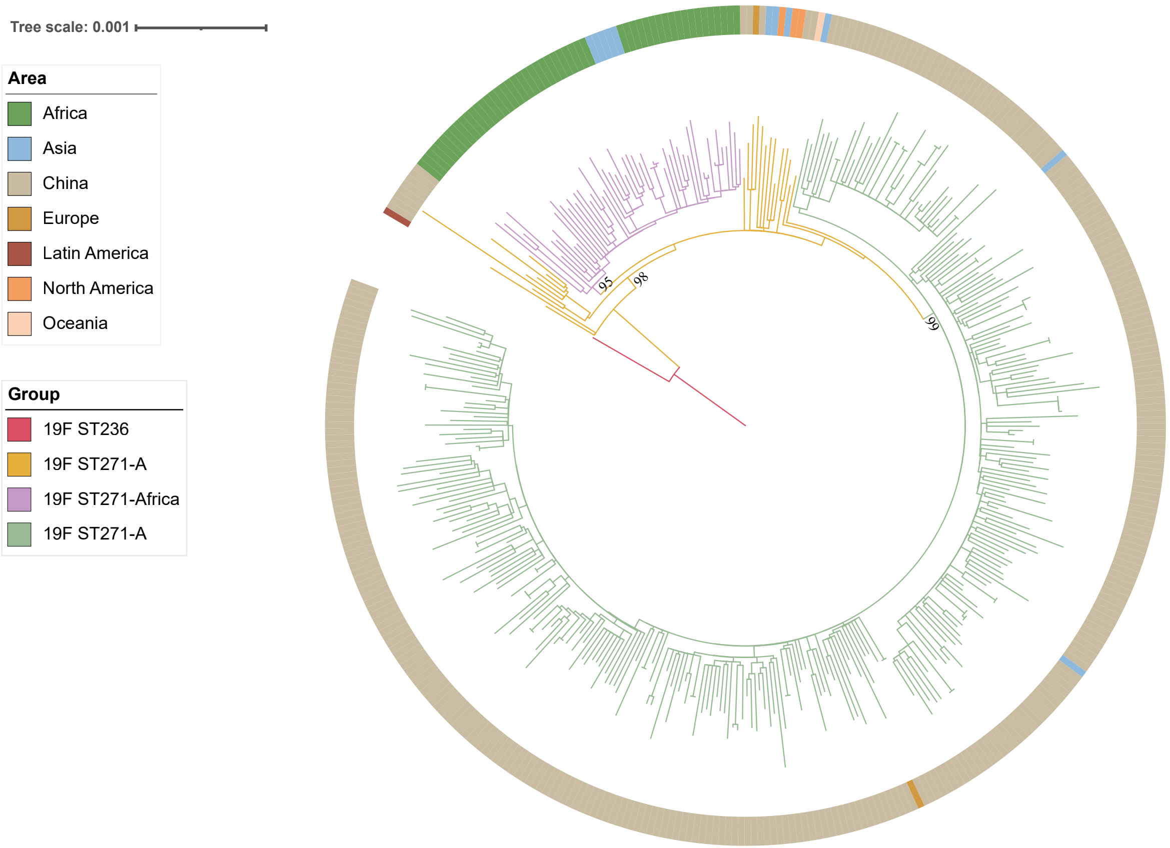

Figure S4. Reconstructed Recombination-masked phylogeny of 387 19F ST271 genomes. Color strip represent the continent or country from which the sample was isolated, "Asia" represents Asian countries other than China. Branch colors correspond to groups. The ultrafast bootstrap values of important nodes are shown.

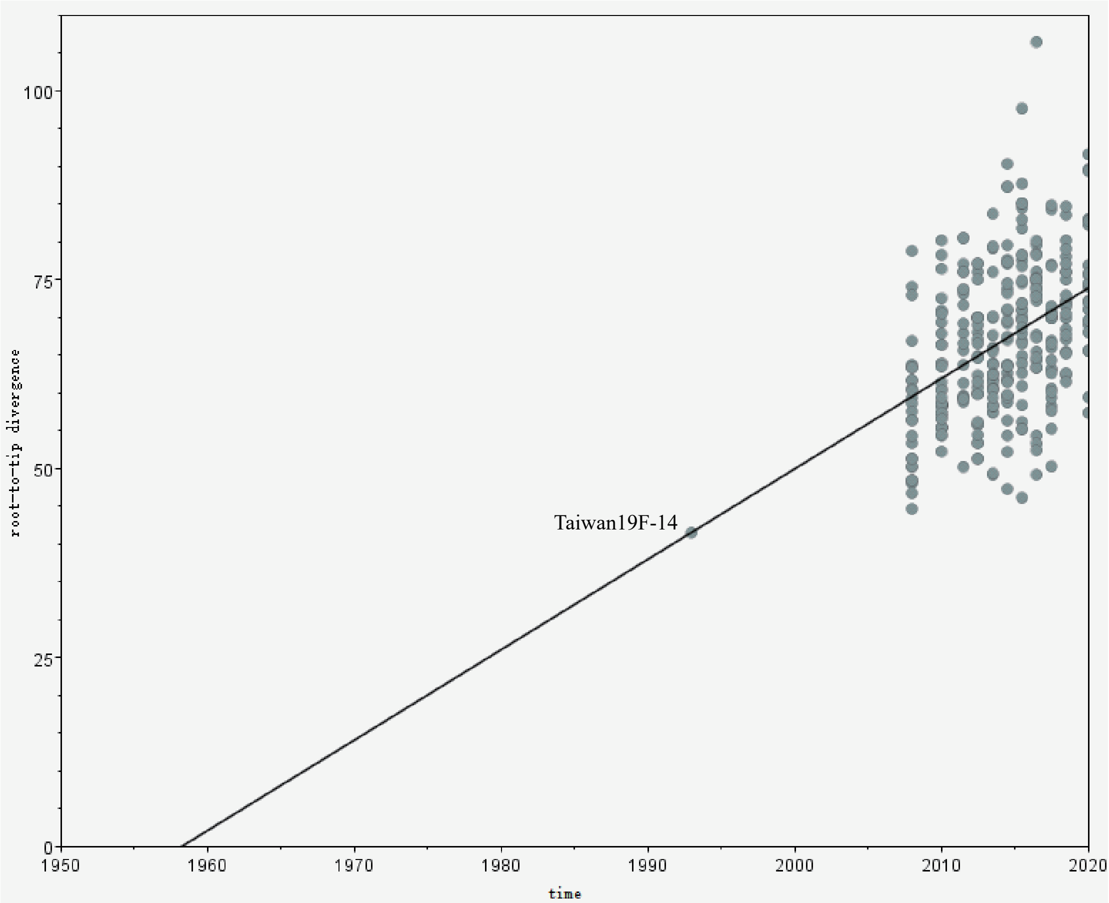

Figure S5. Screenshot of TempEst root-to-tip plot of 301 19F ST271 isolates in China. This dataset exhibit a positive correlation between genetic distance and sampling year ($R^2 = 0.1852$). The Taiwan19F-14 complete genome (NC_012469.1) used as outgroup were marked in the plot.

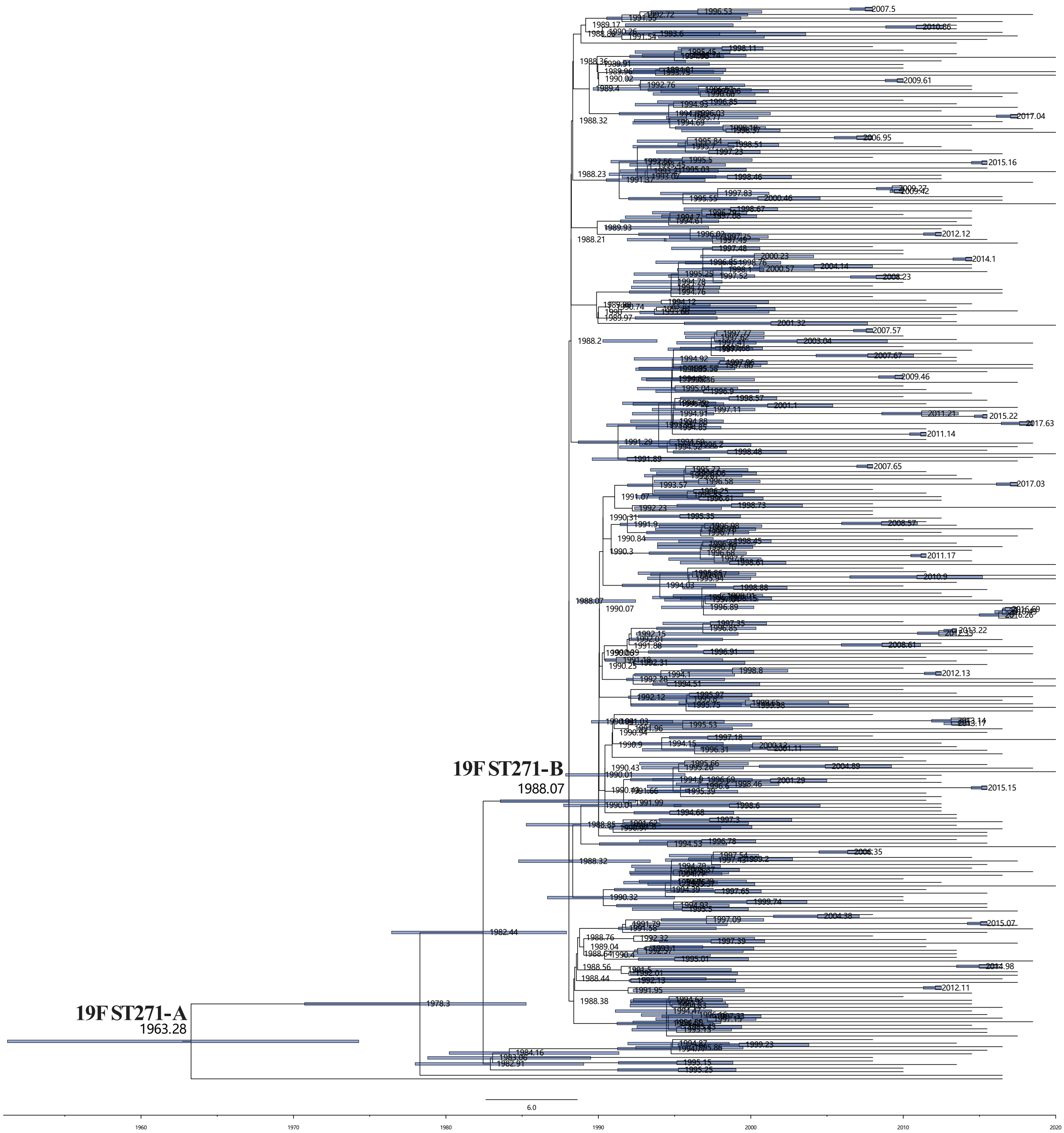

Figure S6. Time-scaled phylogeny of 301 19F ST271 genomes with Bayesian skyline plot as tree prior. Nodes of the respective tMRCA (the most recent common ancestor) of 19F ST271-A and 19F ST271-B are marked in the figure.
